# Transcranial Magnetic Stimulation for Improving Dysphagia After Stroke: A Meta-Analysis of Randomized Controlled Trials

**DOI:** 10.3389/fnins.2022.854219

**Published:** 2022-04-22

**Authors:** Yu-lei Xie, Shan Wang, Jia-meng Jia, Yu-han Xie, Xin Chen, Wu Qing, Yin-xu Wang

**Affiliations:** ^1^Department of Rehabilitation Medicine, Affiliated Hospital of North Sichuan Medical College, Nanchong, China; ^2^North Sichuan Medical College, Nanchong, China; ^3^Department of Rehabilitation Medicine, Chengdu Second People's Hospital, Chengdu, China; ^4^University of South China, Hengyang, China

**Keywords:** deglutition disorders, transcranial magnetic stimulation, stroke, meta-analysis, systematic review

## Abstract

**Background:**

Rehabilitation of post-stroke dysphagia is an urgent clinical problem, and repetitive transcranial magnetic stimulation (rTMS) has been widely used in the study of post-stroke function. However, there is no reliable evidence-based medicine to support the effect of rTMS on post-stroke dysphagia. This review aims to evaluate the effectiveness and safety of rTMS on post-stroke dysphagia.

**Methods:**

English-language literature published before December 20, 2021, were searched in six electronic databases. Identified articles were screened, data were extracted, and the methodological quality of included trials was assessed. Meta-analysis was performed using RevMan 5.3 software. The GRADE method was used to assess the quality of the evidence.

**Results:**

A total of 10 studies with 246 patients were included. Meta-analysis showed that rTMS significantly improved overall swallowing function (standardized mean difference [SMD]−0.76, 95% confidence interval (CI)−1.07 to−0.46, *p* < 0.0001, *n* = 206; moderate-quality evidence), Penetration Aspiration Scale (PAS) (mean difference [MD]−1.03, 95% CI−1.51 to−0.55, *p* < 0.0001, *n* = 161; low-quality evidence) and Barthel index scale (BI) (MD 23.86, 95% CI 12.73 to 34.99, *p* < 0.0001, *n* = 136; moderate-quality evidence). Subgroup analyses revealed that (1) rTMS targeting the affected hemisphere and targeting both hemispheres significantly enhanced overall swallowing function and reduced aspiration. (2) Low-frequency rTMS significantly enhanced overall swallowing function and reduced aspiration, and there was no significant difference between high-frequency rTMS and control group in reducing aspiration (*p* = 0.09). (3) There was no statistical difference in the dropout rate (low-quality evidence) and adverse effects (moderate-quality evidence) between the rTMS group and the control group.

**Conclusion:**

rTMS improved overall swallowing function and activity of daily living ability and reduced aspiration in post-stroke patients with good acceptability and mild adverse effects.

## Introduction

Stroke, as a common cerebrovascular disease, is the primary cause of disability worldwide (Gorelick, 2019). About 19-81% of survivors after stroke are left with dysphagia, which is characterized by varing degree of eating disorders, choking cough, salivation and abnormal pronunciation (Martino et al., 2005; Suntrup et al., 2015). Dysphagia is associated with increased risk of malnutrition and pneumonia, and leads to prolonged hospital stay, poor prognosis and mortality (Park et al., 2017; Pandian et al., 2018; Alamer et al., 2020). Therefore, the rehabilitation of post-stroke dysphagia is still an urgent clinical problem.

Repetitive transcranial magnetic stimulation (rTMS), as a non-invasive neuromodulation technique, is an emerging choice for post-stroke dysphagia (Lefaucheur et al., 2020). In general, rTMS can be divided into two main treatment protocols according to the stimulation frequency: low frequency (≤ 1 Hz) and high frequency (> 1 Hz). Low frequency rTMS (LF-rTMS) inhibits cortical excitability, while high frequency rTMS (HF-rTMS) activates cortical excitability (Lin et al., 2019). It is now recognized that rTMS can inhibit maladaptive cortical plasticity, improve adaptive cortical activity, and promote neurological recovery after stroke (Kobayashi and Pascual-Leone, 2003). According to the latest evidence-based guidelines for rTMS, rTMS has been proved to show the efficacy of A grade in treatment of depression, neuropathic pain, and upper limb dysfunction after stroke (Lefaucheur et al., 2020).

In recent years, several meta-analyses (Yang et al., 2015, 2021; Liao et al., 2017; Lin et al., 2019; Cheng et al., 2021; Li et al., 2021) have investigated the effects of rTMS on post-stroke dysphagia, suggesting that rTMS may have beneficial effects on swallowing disorders. However, some of reviews focused on non-invasive brain stimulation (NIBS), including rTMS, transcranial Direct Current Stimulation (tDCS) and other kinds of stimulation, while few of reviews further analyzed the effects of stimulation site, frequency and stimulation time on dysphagia. A recent meta-analysis (Yang et al., 2021), partially affirming the effects of rTMS on post-stroke dysphagia, concluded in its subgroup analysis of intervention frequency that there was no statistically significant difference between either the high-frequency and low-frequency groups or the conventional training group, which may be related to incorrect data extraction and exclusion of some studies that met their inclusion criteria. A growing body of evidence supports the beneficial effects of transcranial magnetic stimulation on post-stroke dysphagia (Lefaucheur et al., 2020), but the relationship between transcranial magnetic stimulation and factors such as target, parameter settings, and treatment course remains to be further investigated. Therefore, this meta-analysis aims to provide the latest evidence on the effects of transcranial magnetic stimulation on post-stroke swallowing disorders.

## Materials and Methods

This work adhered to the Preferred Reporting Items for Systematic Reviews and Meta-Analyses (PRISMA) guidelines (Ardern et al., 2021).

### Search Strategies

The following databases were searched to identify studies on the effect of rTMS on post-stroke dysphagia, published before December 20, 2021: PubMed, Cochrane Library, ScienceDirect, MEDLINE, and Web of Science for relevant studies. The English keywords used for the database searches were “stroke,” “transcranial magnetic stimulation,” “repetitive transcranial magnetic stimulation,” “TMS,” “rTMS,” “deglutition disorders,” and “dysphagia.” The reference lists of identified articles were checked for other potential studies.

### Inclusion and Exclusion Criteria

Two review authors independently assessed the methodological quality of the included studies. We recorded and resolved any disagreements through discussions with a third reviewer.

Clinical studies that meet the following criteria were included:

(1) All patients with ischemic or hemorrhagic stroke displayed definitive radiographic evidence of relevant pathology on magnetic resonance imaging (MRI) or computed tomography (CT);

(2) All participants were identified as having dysphagia;

(3) No participants had swallowing disorders caused by other diseases;

(4) Randomized controlled trials compared rTMS with sham stimulation or other routine rehabilitation training.

If data were repeated or shared in multiple studies, the study that best met the above criteria were considered. All published or unpublished studies were investigated. If the information required for the analysis could not be obtained from the publication, the author was contacted to obtain the necessary details.

### Risk of Bias and Quality of Outcomes Assessment

Two review authors independently assessed the methodological quality of the included studies. A third reviewer recorded and resolved any disagreements. Each RCT used Cochrane's collaborative tools to assess the risk of bias, including adequacy of sequence generation, concealment of allocation, blinding of participants and personnel, blinding of result evaluators, incomplete results' data, selective reporting, and other biases (Higgins et al., 2011; Corbett et al., 2014). The Grading of Recommendations Assessment, Development and Evaluation (GRADE) guidelines for systematic reviews were used to evaluate the quality of outcomes (Guyatt et al., 2008).

### Data Extraction

All searches and included studies were conducted by two independent reviewers. If there was any objection, a third reviewer made the final decision. The following data were extracted from the final included researches: basic study information (study authors, year of publication), participant characteristics (age, and sample size), rTMS parameters [stimulus site, true stimulus frequency, stimulus intensity (% of motor threshold (MT)), and treatment regimen], overall swallowing function and activity of daily living outcome measures, dropout rate, and adverse effects.

### Outcome Indicators

Outcome measures for the efficacy of therapy were as follows: (1) DD (Dysphagia Grade); (2) Functional Dysphagia Scale (FDS); (3) Videofluoroscopic Dysphagia Scale (VDS); (4) Penetration Aspiration Scale (PAS); (5) Barthel index scale (BI); (6) dropout rate; (7) adverse effects.

The DD is a four-level score for the swallowing function according to patients' clinical manifestations (Khedr and Abo-Elfetoh, 2010). The FDS is a scale quantifying dysphagia severity (Han et al., 2001). The VDS, with a sum of 100 points, is a reliable, objective, and quantifiable predictor of long-term dysphagia after stroke (Kim et al., 2014). The PAS is an 8 point multidimensional indicator of airway invasion that measures selected aspects such as penetration and inhalation, depth of invasion into the delivery airway, and whether substances entering the airway are expelled (Martin-Harris et al., 2005). The higher the score of the above 4 scales, the worse the swallowing function. If dysphagia outcomes were reported from multiple time points, those from immediately after the intervention were obtained for meta-analysis.

### Statistical Analyses

All statistical analysis used the RevMan 5.3 statistical software (The Nordic Cochrane Center, The Cochrane Collaboration, Copenhagen, Denmark), and the heterogeneity of different research results was tested by the overlap of confidence intervals and chi-square tests. When there was no heterogeneity in the test results, fixed-effect model was used for the meta-analysis, and when the test results were heterogeneous, the random-effect model was used. For enumeration data, the risk ratio (RR) and 95% confidence intervals (CIs) were used as the statistical tool for the efficacy analysis and the effect size, respectively. If substantial heterogeneity was detected (*I*^2^ > 50%), subgroup analysis or sensitivity analysis was conducted to determine the source of heterogeneity.

## Results

### Search and Selection of Studies

**Figure 1:**
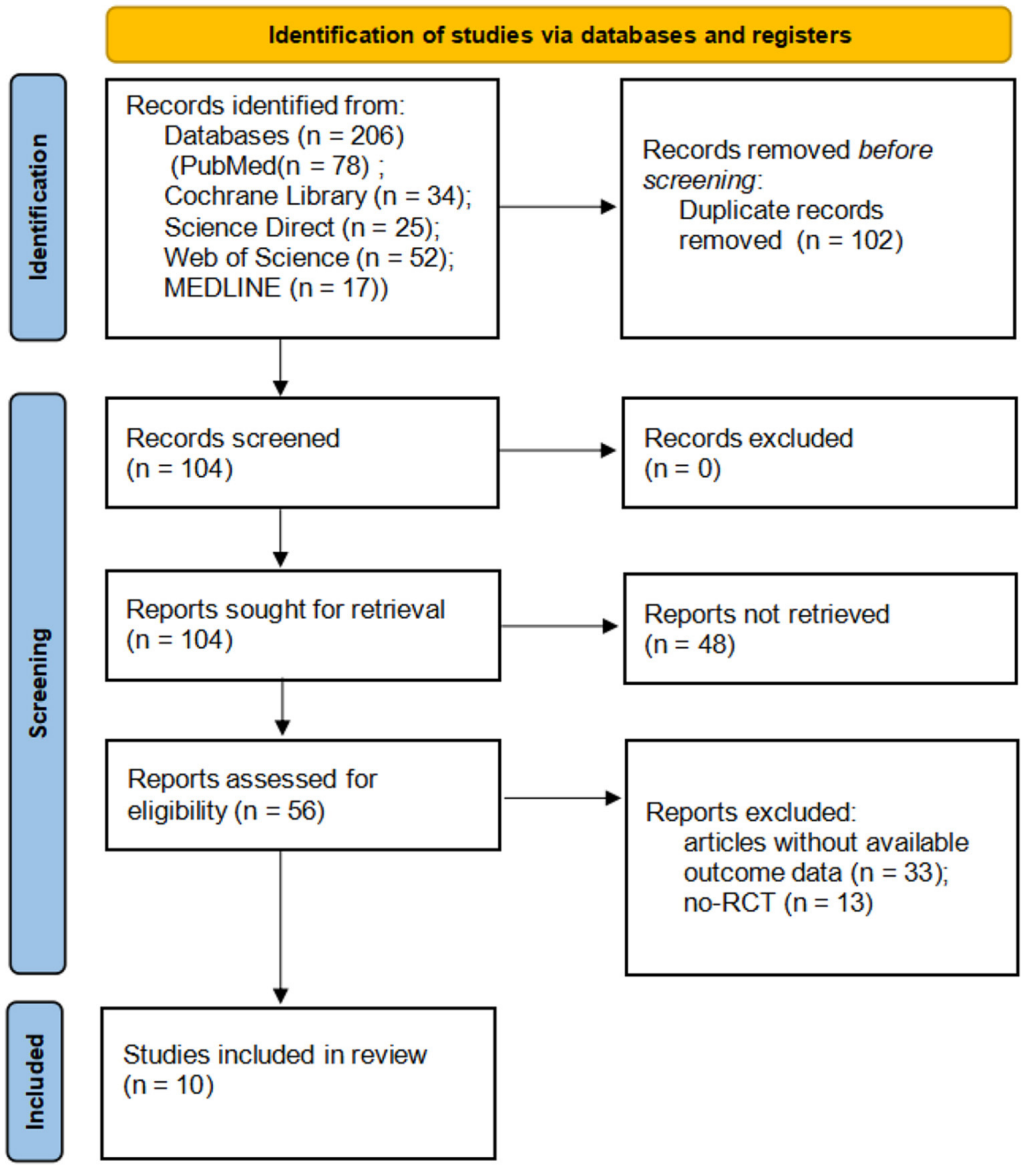
PRISMA flowchart of the study selection process. randomized controlled trials.

The study selection process is shown in Figure 1. A total of 206 potential relevant studies were screened from six English-language databases using a relevant search strategy. Of these relevant studies, 102 duplicates were removed and the remaining 104 studies were further evaluated for eligibility. An additional 48 articles were removed after screening the title and abstract. Finally, after reviewing the full text of the remaining 56 articles, 46 articles were excluded, and a total of 10 studies were included.

### Characteristics of the Included Studies

Table 1 shows the characteristics of the 10 studies included in this meta-analysis with a total of 246 participants (149 in the rTMS group and 109 in the control group). Participants all were identified dysphagia according to either the videofluoroscopic swallowing study (VFSS) or Fiberoptic endoscopic evaluation of swallowing (FEES). Four studies (Lim et al., 2014; Tarameshlu et al., 2019; Unluer et al., 2019; Cabib et al., 2020) used LF-rTMS; 4 studies (Khedr et al., 2009; Khedr and Abo-Elfetoh, 2010; Park et al., 2013, 2017) used HF-rTMS; and 2 studies (Kim et al., 2011; Du et al., 2016) compared the efficacy of LF-rTMS and HF-rTMS. rTMS stimulation sites included the ipsilesional hemisphere, the contralesional hemisphere, and the bilateral hemisphere. The interventions of control group included sham rTMS stimulation among 7 studies (Khedr et al., 2009; Khedr and Abo-Elfetoh, 2010; Kim et al., 2011; Park et al., 2013, 2017; Du et al., 2016; Cabib et al., 2020), and 3 studies (Lim et al., 2014; Tarameshlu et al., 2019; Unluer et al., 2019) with conventional therapy.

**Table 1:** Characteristics of the randomized controlled studies.

**References**	**Intervention**	**Age (M ±SD)**	**Sample size (M/F)**	**Site for stimulation (esophageal cortical)**	**Stimulation parameters**	**Outcomes/measure**
Khedr et al. (2009)	Active rTMS	58.9 (11.7)	14	The ipsilesional hemisphere, esophageal motor cortex;	3 Hz, 120%MT, 10 min, 5 times	DD, BI, MEP, dropout rate
	Sham rTMS	56.2 (13.4)	12			
Khedr and Abo-Elfetoh (2010)	Active rTMS	LMI: 56.7 (16)	6/0	The bilateral hemisphere, esophageal motor cortex;	3 Hz, 130%MT, 10 min, 5 times	DD, BI
		Other: 55.4 (9.7)	2/3			
	Sham rTMS	LMI: 58 (17.5)	5/0			
		Other: 60.5 (11)	3/3			
Kim et al. (2011)	Sham rTMS	68.2 (12.6)	6/4	The ipsilesional hemisphere, mylohyoid motor cortex;	5 Hz, 100%MT, 20 min, 10 times	FDS, PAS, ASHA NOMS
	High-frequency rTMS	69.8 (8.0)	5/5			
	Low-frequency rTMS	66.4 (12.3)	6/4	The contralesional side, mylohyoid motor cortex;	1 Hz, 100%MT, 20 min, 10 times	
	Sham rTMS	68.9 (9.3)	5/4			
Lim et al. (2014)	Active rTMS	62.5 (8.2)	14	The contralesional side, pharyngeal motor cortex;	1 Hz, 100%MT, 20 min, 10 times	FDS, PAS, PTT, ASHA NOMS, adverse effects, dropout rate
	Conventional dysphagia therapy	59.8 (11.8)	15			
Park et al. (2017)	Sham rTMS	69.6 (8.6)	7/4	The bilateral hemisphere, mylohyoid motor cortex;	10 Hz, 90%MT, 10 min, 10 times	VDS, PAS
	Bilateral rTMS	60.2 (13.8)	8/3			
	Unilateral rTMS	67.5 (13.4)	8/3	The ipsilesional hemisphere, mylohyoid motor cortex;		
Du et al. (2016)	Sham rTMS	58.83 (3.35)	6/6	The ipsilesional hemisphere, mylohyoid motor cortex;	3 Hz, 90%MT, 1200 pulses, 5 times	SSA, BI, DD, adverse effects, dropout rate
	High-frequency rTMS	58.2 (2.78)	13/2			
	Low-frequency rTMS	57.92 (2.47)	7/6	The contralesional side, mylohyoid motor cortex;	1 Hz, 100%MT, 1200 pulses, 5 times	
	Conventional dysphagia therapy	69.31 (12.89)	7/6			
Tarameshlu et al. (2019)	Active rTMS	55.33 (19.55)	4/2	The contralesional side, mylohyoid motor cortex;	1 Hz, 120%MT, 1200 pulses, 5 times	MASA, FOIS
	Conventional dysphagia therapy	76.67 (5.92)	5/1			
Cabib et al. (2020)	Active rTMS	70 (8.6)	12	The contralesional side, pharyngeal sensory cortex;	5 Hz, 90%MT, 250 pulses, 1 time	PAS, MEP, adverse effects
	Sham rTMS	70 (8.6)	12			

*BI, Barthel Index Scale; DD, The degree of dysphagia; MEP, motor-evoked potential; LMI, Lateral medullary infarction; FDS, the Functional Dysphagia Scale; PAS, the Penetration Aspiration Scale; ASHA NOMS, the American Speech-Language Hearing Association National Outcomes Measurements System Swallowing Scale; VDS, the videofluoroscopic dysphagia scale; PTT, the pharyngeal transit time; SSA, the Standardized Swallowing Assessment; MASA, the Mann Assessment of Swallowing Ability; FOIS, the Functional Oral Intake Scale; IPES, intra-pharyngeal electrical stimulation*.

In terms of outcome measures, different dysphagia measurement tools were used to assess swallowing function within the same study or between studies. Overall swallowing function measures included DD [4 studies (Khedr et al., 2009; Khedr and Abo-Elfetoh, 2010; Du et al., 2016; Tarameshlu et al., 2019)], FDS [2 studies (Kim et al., 2011; Lim et al., 2014)], VDS [2 studies (Park et al., 2013, 2017)]. Aspiration was assessed by PAS [6 studies (Kim et al., 2011; Park et al., 2013, 2017; Lim et al., 2014; Unluer et al., 2019; Cabib et al., 2020)]. BI was used to assess activity of daily living.

### Research Quality

**Figure 2:**
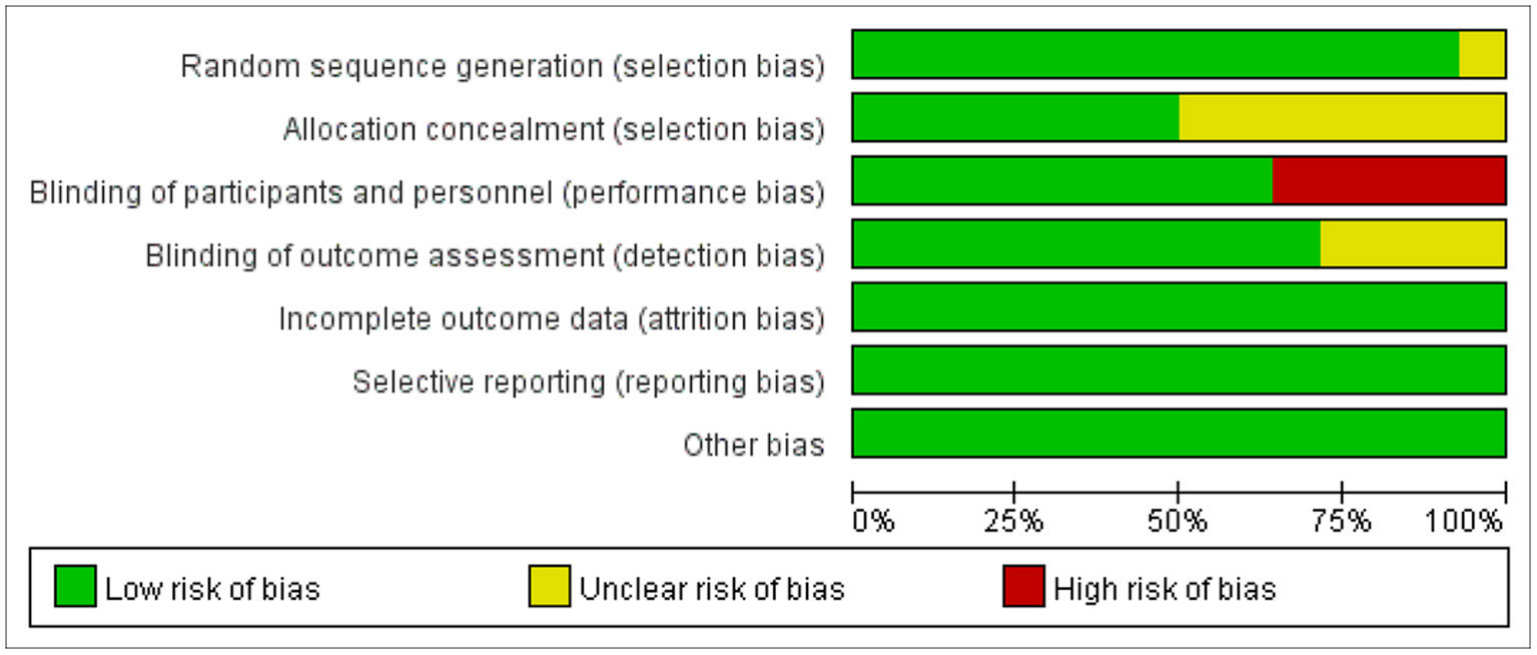
Performance of each type of bias in all studies.

In all included literature, some of articles designed two experimental groups based on parameters such as lesion site and stimulation frequency. According to this review, the two experimental groups did not interfere with each other in the same literature. Therefore, we treated each study in these three articles as a randomized controlled experiment. There was also one study that divided the patients into two randomized controlled trials based on the site of the disease, and we combined and merged the data. We selected 13 studies from 10 articles. Two researchers assessed the quality of the 13 included studies. Data completeness was assured in a large extent, but 4 studies (Park et al., 2013; Lim et al., 2014; Unluer et al., 2019; Cabib et al., 2020) had performance bias (complete blinding of subjects was not achieved) (Figure 2). The number of this meta-analysis included is very small, so we could not use funnel plots to assess publication bias. Therefore, publication bias could not be completely eliminated.

### Meta-Analysis of Treatment Effect

#### Overall Swallowing Function

**Figure 3:**
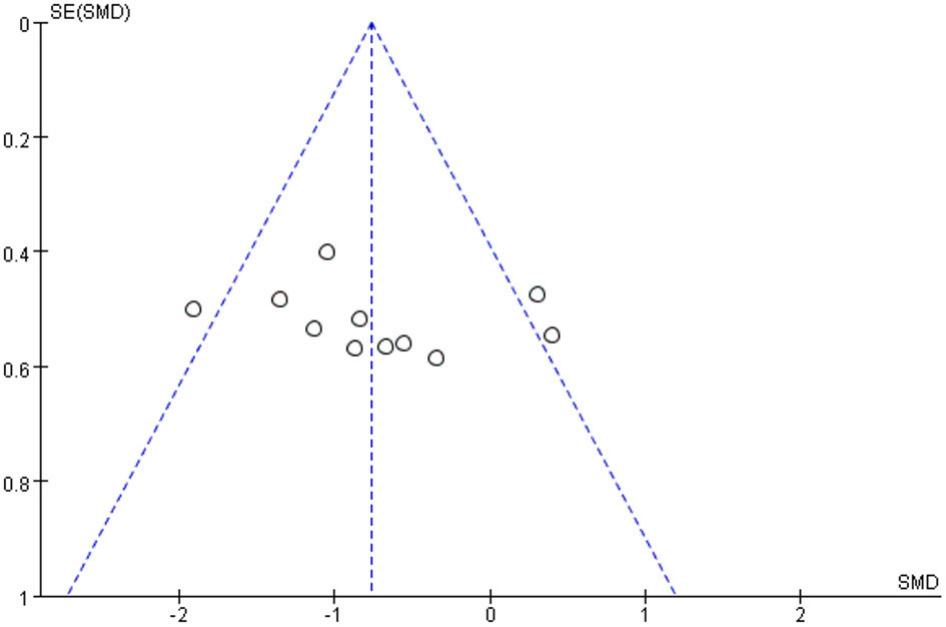
Funnel plot for the publication bias of overall swallowing function.

**Figure 4:**
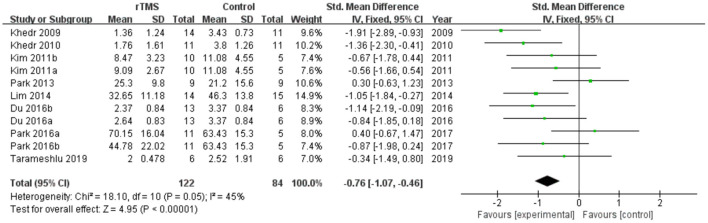
Forest plot for overall swallowing function.

Ten studies involving a total of 206 patients with post-stroke dysphagia evaluated the effect of rTMS on overall swallowing function. Heterogeneity of included studies was low (*I*^2^ = 45%), and therefore a fixed-effect model was used for meta-analysis. The funnel plot revealed significant symmetry (Figure 3). The simulated results showed that the rTMS significantly improved overall swallowing function compared to the control group (standard mean difference [SMD]−0.76, 95% confidence interval (CI)−1.07 to−0.46, *p* < 0.0001) (Figure 4). According to the GRADE, the overall level of evidence for the effect of rTMS on global swallowing function was “Moderate” (Table 2).

**Table 2:** GRADE quality of evidence assessment of individual outcome indicators for the efficacy of repetitive transcranial magnetic stimulation in the treatment of dysphagia.

**Outcome indicator**	**Number of participants**	**Heterogeneity**		**Model of analysis**	**Group effect value**		**Estimated value**	**95% CI**	**Grade**
		***I* ^2^**	***P***		**Z**	***P***			
Overall swallowing function	206 (11 RCT)	45%	0.05	Fixed effect	4.95	<0.0001	−0.76 (SMD)	−1.07,−0.46	Moderate
PAS	161 (8 RCT)	23%	0.24	Fixed effect	4.18	<0.0001	−1.03 (MD)	−1.51,−0.55	Low
BI	85 (3 RCT)	0%	0.89	Fixed effect	4.2	<0.0001	23.86 (MD)	12.73, 34.99	Moderate
Dropout rate	136 (4 RCT)	0%	0.53	Fixed effect	0.33	0.74	0.87 (RR)	0.38, 2.00	Low
Adverse effects	121 (4 RCT)	0%	0.91	Fixed effect	1.41	0.16	2.61 (RR)	0.69, 9.86	Moderate

*RCT, randomized controlled trials; SMD, standardized mean difference; RR, relative risk; CI, confidence interval; PAS, Penetration Aspiration Scale; BI, Barthel index scale; GRADE, Grading of Recommendation Assessment, Development and Evaluation*.

#### Subgroup Analysis of Overall Swallowing Function

**Figure 5:**
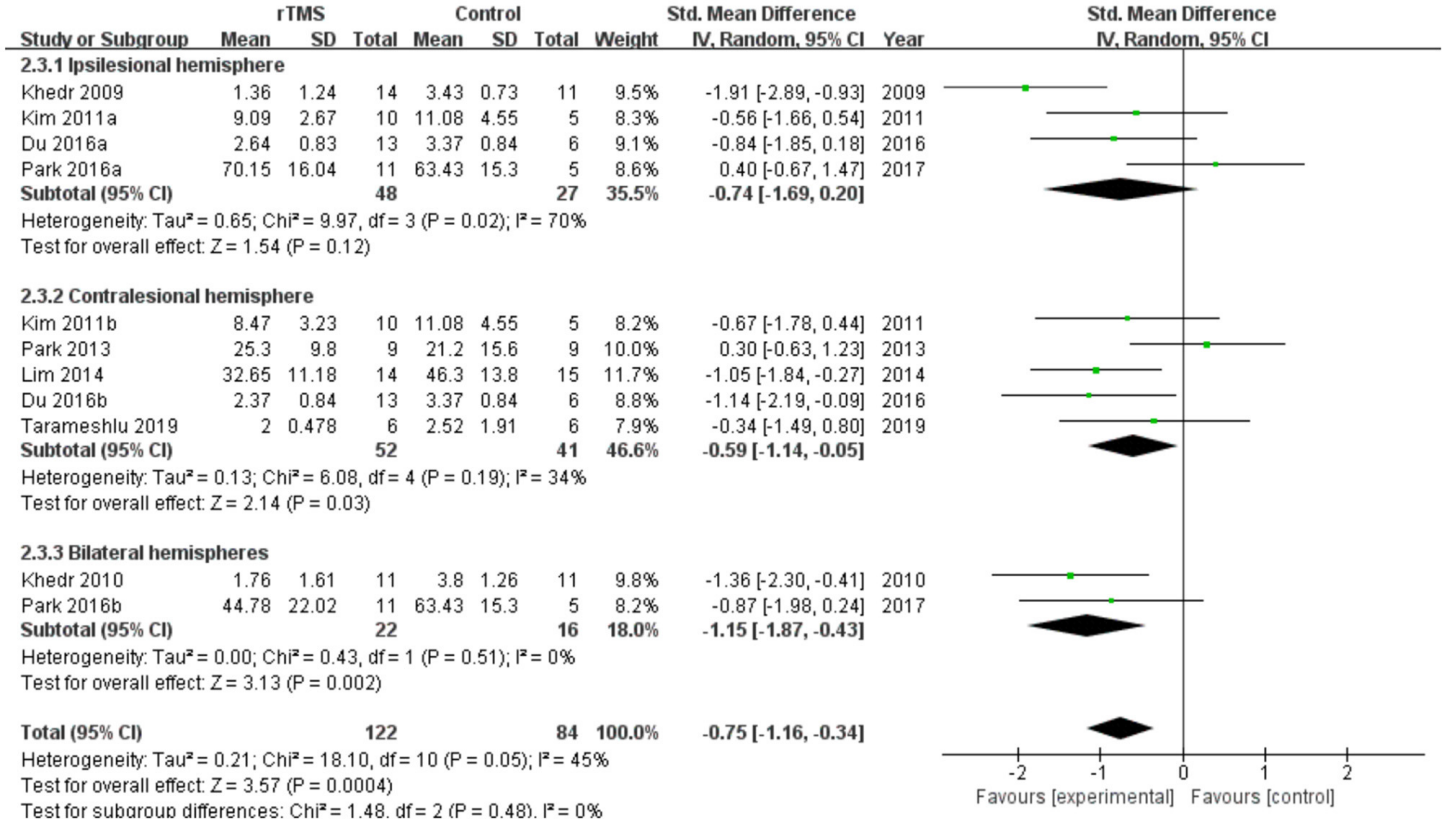
Forest plot for subgroup analysis for overall swallowing function: ipsilesional hemisphere vs. contralesional hemisphere vs. bilateral hemispheres.

**Figure 6:**
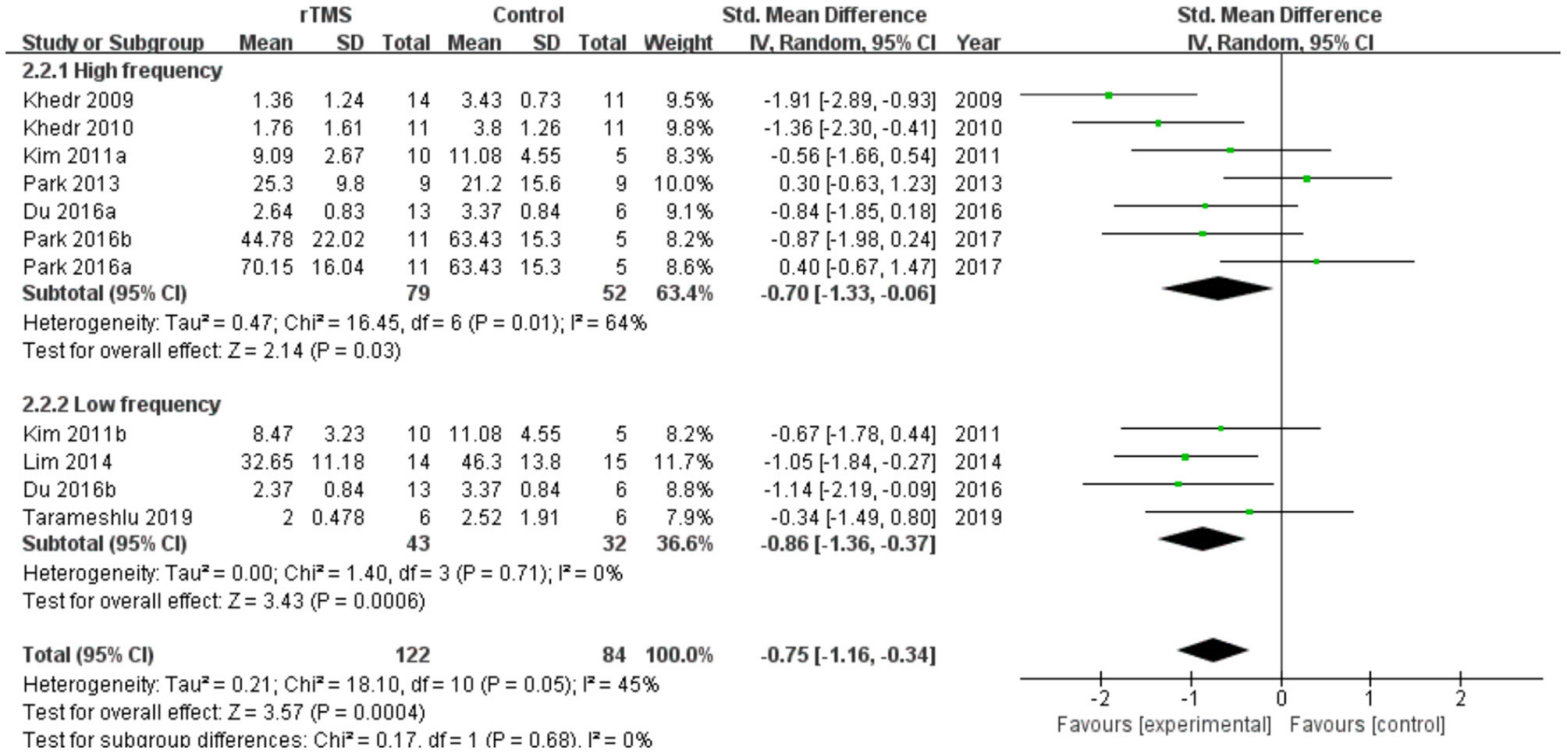
Forest plot for subgroup analysis for overall swallowing function: low frequency transcranial magnetic stimulation vs. high frequency transcranial magnetic stimulation.

Subgroup analyses were performed according to stimulus site (the ipsilesional hemisphere, the contralesional hemisphere, and the bilateral hemisphere). Subgroup analysis showed that the SMD for trials involving the “the ipsilesional hemisphere” stimulus was−0.74 (95% CI−1.69 to 0.20, *p* = 0.12) and for trials involving the “the contralesional hemisphere” stimulus was−0.59 (95% CI−1.14 to−0.05, *p* = 0.03). The mean effect size for trials involving “the bilateral hemisphere” stimulus was−1.15 (95% CI−1.87 to−0.43) (Figure 5). Stimulation of the bilateral hemisphere may produce better therapeutic effects on overall swallowing function. Subgroup analyses were performed according to stimulation frequency (LF-rTMS, HF-rTMS). Subgroup analysis showed a SMD of−0.70 (95% CI−1.33 to -−0.06) for the studies of HF-rTMS. The study of LF-rTMS showed a SMD of−0.86 (95% CI−1.16 to - 0.34). These results suggested that LF-rTMS treatment produced better effects on overall swallowing function than HF-rTMS treatment (Figure 6).

### PAS

**Figure 7:**
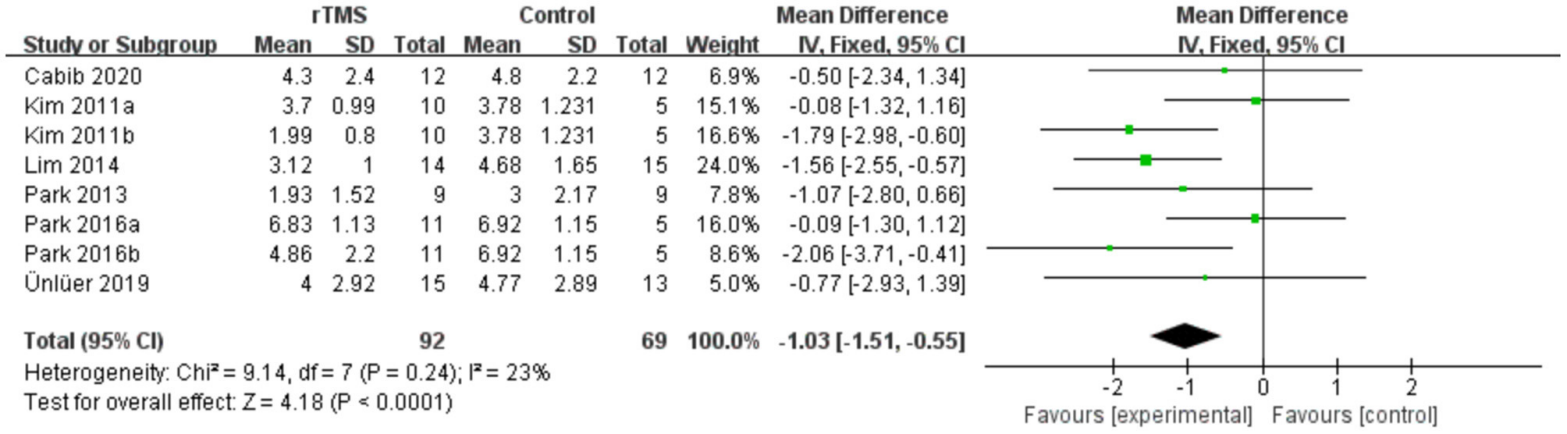
Forest plot for subgroup analysis for Penetration Aspiration Scale.

Seven studies involving a total of 161 patients with post-stroke dysphagia evaluated the effect of rTMS on PAS. Heterogeneity of included studies was low (*I*^2^ = 23%), and therefore a fixed-effect model was used. The simulated results showed that the rTMS significantly reduced accidental aspiration compared to the control group (mean difference [MD]−1.03, 95% CI−1.51 to−0.55, *p* < 0.0001) (Figure 7). According to the GRADE, the overall level of evidence for the effect of rTMS on PAS was “Low” (Table 2).

#### Subgroup Analysis of PAS

**Figure 8:**
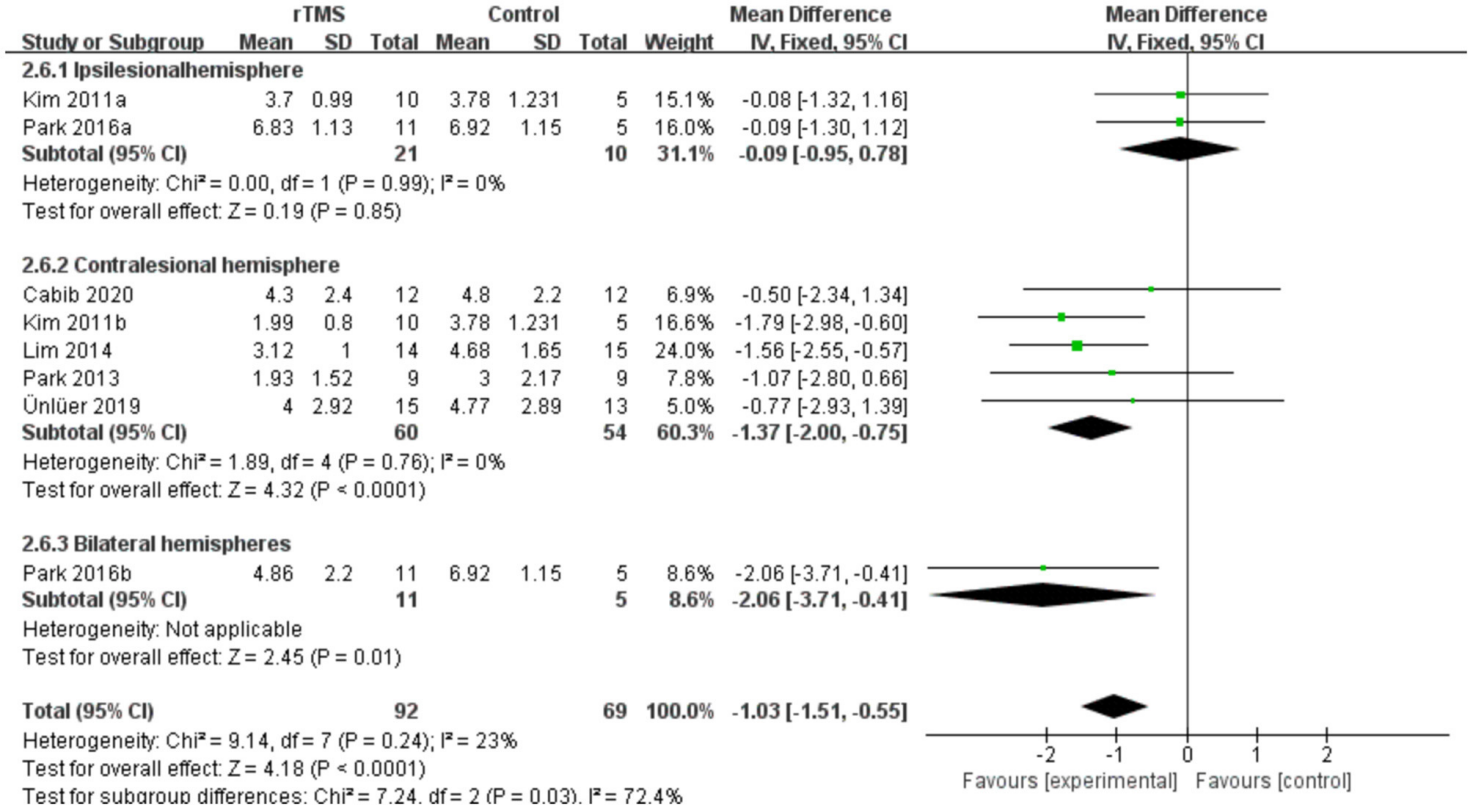
Forest plot for subgroup analysis for Penetration Aspiration Scale: ipsilesional hemisphere vs. contralesional hemisphere vs. bilateral hemispheres.

**Figure 9:**
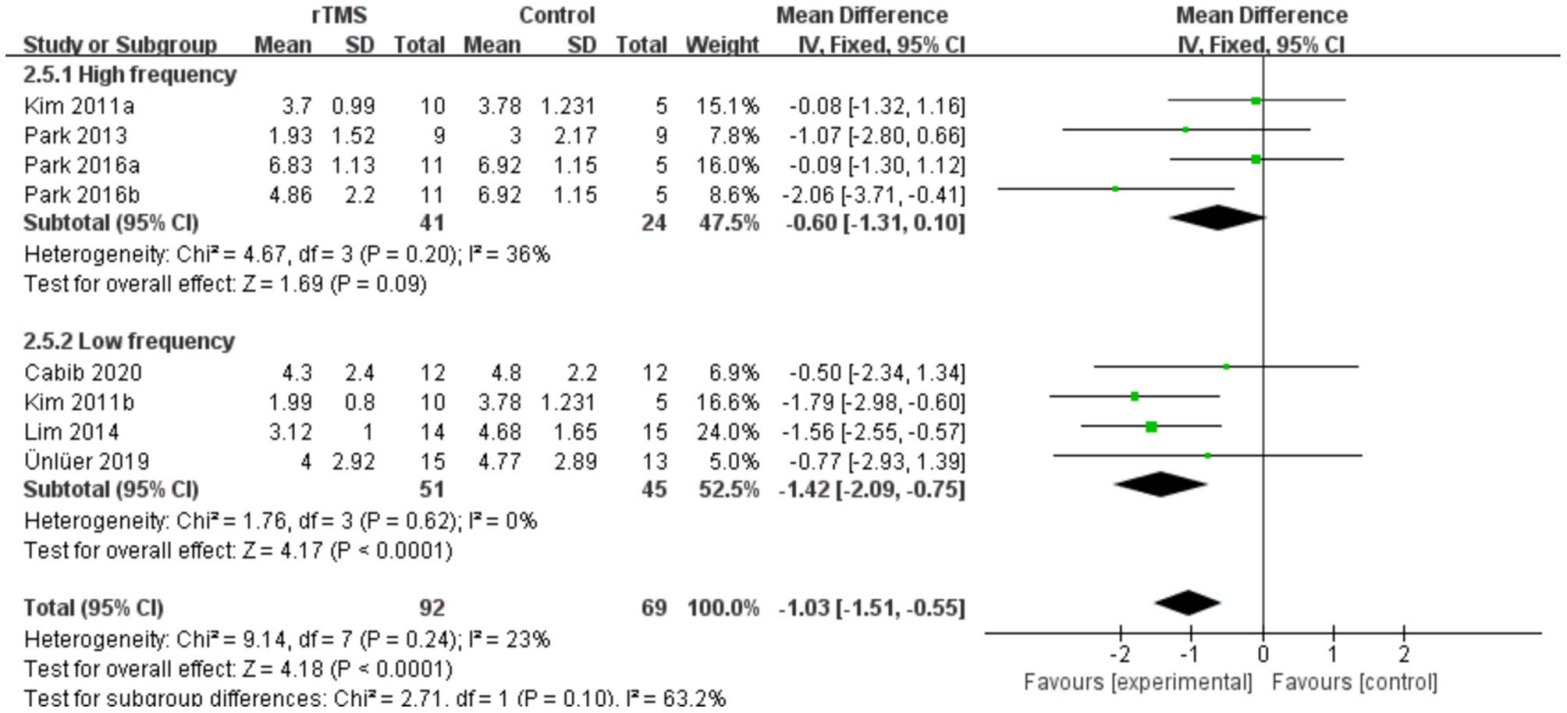
Forest plot for subgroup analysis for Penetration Aspiration Scale: low frequency Transcranial Magnetic Stimulation vs. high frequency Transcranial Magnetic Stimulation.

Subgroup analyses were performed according to stimulus site (the ipsilesional hemisphere, the contralesional hemisphere, and the bilateral hemisphere). Subgroup analysis showed that the MD for trials involving the “the ipsilesional hemisphere” stimulus was−0.09 (95% CI−0.95 to 0.78, *p* = 0.85) and for trials involving the “the contralesional hemisphere” stimulus was−1.37 (95% CI−2.00 to−0.75, *p* < 0.0001). The MD for trials involving “the bilateral hemisphere” stimulus was−2.06 (95% CI−3.71 to−0.41) (Figure 8). Stimulation of the bilateral hemisphere may produce better therapeutic effects on overall swallowing function. Subgroup analyses were performed according to stimulation frequency (LF-rTMS, HF-rTMS). Subgroup analysis showed a MD of−0.60 (95% CI−1.31 to -−0.10) for the studies of HF-rTMS. The studies of LF-rTMS showed a SMD of−1.42 (95% CI−2.09 to - 0.75). These results suggest that LF-rTMS treatment produced better effects on overall swallowing function than HF-rTMS treatment (Figure 9).

### BI

**Figure 10:**
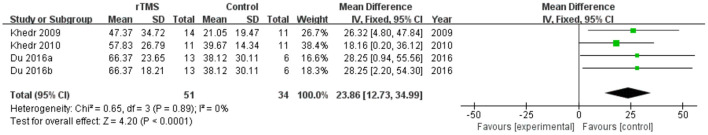
Forest plot for subgroup analysis for Barthel index scale.

Four studies involving a total of 137 patients with post-stroke dysphagia evaluated the effect of rTMS on BI. Heterogeneity of included studies was low (*I*^2^ = 0%), and therefore a fixed-effect model was used for meta-analysis. The simulated results showed that the rTMS significantly improved activity of daily living compared to the control group (MD 23.86, 95% CI 12.73 to 34.99, *p* < 0.0001) (Figure 10). According to the GRADE, the overall level of evidence for the effect of rTMS on BI was “Moderate” (Table 2).

### Meta-Analysis of Dropout Rate

**Figure 11:**
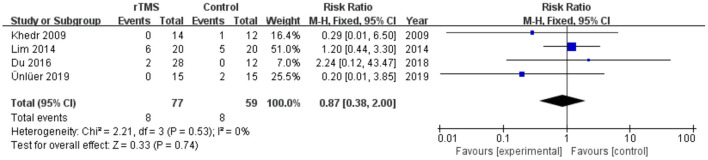
Forest plot for subgroup analysis for Dropout Rate.

Four studies involving a total of 136 patients with post-stroke dysphagia evaluated the effect of rTMS on dropout rate. Heterogeneity of included studies was low (*I*^2^ = 0%), and therefore a fixed-effect model was used for meta-analysis. The results showed no differences in dropout rate between the rTMS group and the control group (RR 0.87, 95% CI 0.38 to 2.00, *p* = 0.74) (Figure 11). According to the GRADE, the overall level of evidence for the effect of rTMS on dropout rate was “Low” (Table 2).

### Meta-Analysis of Adverse Effects

**Figure 12:**
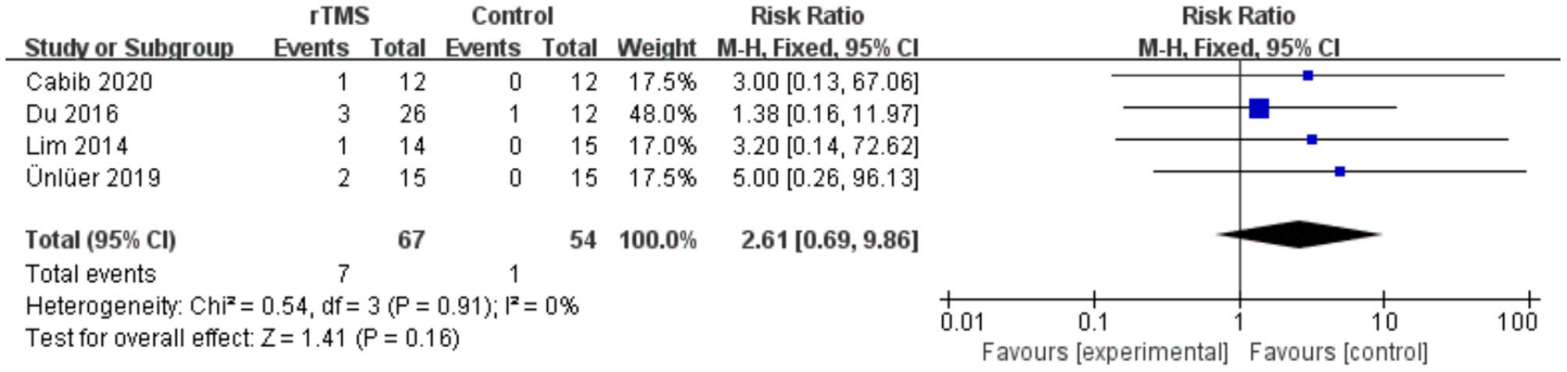
Forest plot for subgroup analysis for Adverse Effects.

No serious adverse reactions were reported in any of the included studies. Four studies reported minor adverse reactions. Seven of 67 patients in the rTMS group and 1 of 54 patients in the control group reported discomfort. Other adverse reactions included headache, dizziness, pain at the site of irritation, and tinnitus. There was no heterogeneity between studies (*I*^2^ = 0%). The results showed no differences in adverse effects between the rTMS group and the control group (RR 2.61, 95% CI 0.69 to 9.86, *p* = 0.16) (Figure 12). According to the GRADE, the overall level of evidence for the effect of rTMS on adverse effects was “Moderate” (Table 2).

## Discussion

This meta-analysis identified 10 studies including a total of 246 patients with post-stroke dysphagia, 149 of whom received 5 to 10 sessions of active rTMS and 109 of whom received sham rTMS or swallowing training. Overall, the results of our meta-analysis supported the benefits of rTMS on overall dysphagia function (moderate-quality evidence) and which reduced instances of aspiration (low-quality evidence) and improved activity of daily living (moderate-quality evidence) for patients with post-stroke dysphagia. rTMS was found to be safe and have no serious adverse effects reported.

Our meta-analysis suggested that rTMS improved swallowing function in post-stroke patients and the heterogeneity of all outcome indicators remained small (*I*^2^ <50%). The funnel plot was symmetrical, suggesting no publication bias in the included studies. The pooled results were generally consistent with previous reviews (Liao et al., 2017; Cheng et al., 2021; Wang et al., 2021), which reported a positive effect of rTMS on recovery from post-stroke dysphagia. The difference from the above studies was that we included only studies in which rTMS was compared with sham stimulation or conventional swallowing treatment, excluding the effect of other types such as NIBS on post-stroke dysphagia.

To reduce potential heterogeneity, we further performed subgroup analysis based on stimulation site and stimulation frequency. Stimulation location subgroup analysis showed that rTMS of the bilateral hemisphere and the contralesional hemisphere significantly improved swallowing function after stroke. In contrast, rTMS of the ipsilesional hemisphere produced lower effect values and the results of the meta-analysis suggested that stimulation of the ipsilesional hemisphere was ineffective, in agreement with the results of the meta-analysis by Liao et al. (2017) and Cheng et al. (2021). Momosaki et al. (2014) reported that 3 Hz rTMS of the bilateral pharyngeal motor cortex resulted in significant recovery on post-stroke dysphagia. Tarameshlu et al. (2019) and Unluer et al. (2019) found that rTMS was effective in improving post-stroke dysphagia and swallowing coordination after stimulation of the unaffected hemisphere. In a randomized controlled trial (Park et al., 2017), Park et al. showed no effect of high-frequency rTMS of the affected hemisphere on swallowing function. However, Du et al. (2016) observed a positive effect of rTMS of the affected hemisphere on swallowing disorders. Therefore, more studies are needed to further validate the effects of rTMS on the affected hemisphere.

Within-frequency subgroup analysis showed that LF-rTMS produced greater effect values than HF-rTMS, suggesting that LF-rTMS is more effective than HF-rTMS on swallowing disorders, which is consistent with the results of Cheng et al. (2021). Kim et al. (2011) conducted a RCT comparing LF-rTMS and HF-rTMS in improving post-stroke dysphagia and found that the effect of LF-rTMS was significant compared with HF-rTMS. In contrast, a meta-analysis by Liao et al. (2017) concluded that the effect size of the HF-rTMS subgroup was greater than that of the LF-rTMS subgroup. This may be related to its early publication and the inclusion of only six studies. Also, we found that the results of our subgroup analysis were not fully consistent with the results of some meta-analyses. Yang et al. (2021) found a simulated effect size SMD = 0.65 (95% CI = 0.04 - 1.26, *p* = 0.04) for rTMS, suggesting that rTMS treatment was superior to conventional treatment. However, subgroup analysis showed no statistical difference between LF-rTMS and HF-rTMS and the conventional training group. This is slightly different from our results, that rTMS showed a significant improvement in overall swallowing function in our pooled analysis, and was also effective in both groups in the subgroup analysis. This may be related to the fact that the review by Yang et al. missed some of the studies that met their inclusion criteria.

Recovery of impaired swallowing function after stroke is complex. Functional magnetic resonance imaging studies suggest that swallowing function may be associated with primary motor sensory cortex, insula, cingulate gyrus, prefrontal cortex, temporal lobe and occipital areas (Mihai et al., 2014, 2016). After stroke, if the injury involves the cortical brainstem tract, medulla oblongata reticular structure or nerve nucleus, the swallowing muscles will not work properly, thus affecting swallowing function (Wilmskoetter et al., 2020). The corticomedullary is the bridge between the brainstem and the swallowing cortex, and a study by Michou et al. confirmed that increased excitability of the corticomedullary was associated with improved swallowing safety (Mihai et al., 2014). Hamdy et al. shown the human swallowing system is bilaterally innervated and is asymmetric (Hamdy et al., 1996). The bilateral cerebral hemispheres maintain normal swallowing function by inhibiting homeostasis through the interaction of the corpus callosum (Lefaucheur et al., 2020). Hamdy et al. also suggested that reorganization of the contralateral pharyngeal cortex was associated with recovery of swallowing function, which demonstrates the role of intact hemispheric reorganization in the recovery of swallowing function after stroke (Hamdy et al., 1998; Fraser et al., 2002).

Therefore, different stimulation protocols will improve post-stroke swallowing disorders through different pathways. First, in unilateral cortical stimulation protocols, current mainstream studies are generally based on the interhemispheric inhibition model, such as Tarameshlu et al. (2019) and Khedr and Abo-Elfetoh (2010) included in our meta-analysis. This theory suggests that the damaged hemisphere decreases excitatory output after brain injury, while the unaffected hemisphere produces excessive inhibition on the affected hemisphere, resulting in various functional impairments (Alia et al., 2017). LF-rTMS stimulates the contralesional hemisphere to produce a long-term depression effect or HF-rTMS stimulates the ipsilesional hemisphere to produce a long-term potentiation effect, thus bringing the rebalance. The long term potentiation effect of HF-rTMS of the ipsilesional hemisphere can bring the imbalanced cortical excitability back to balance, thus improving the function (Lefaucheur et al., 2020). This theory has been widely applied to various types of NIBS, but it can only partially explain the results obtained from our subgroup analysis.

Again, in the compensatory model it was noted that the recovery of dysfunction after brain injury may be related to compensatory reorganization in the unaffected hemisphere, and stimulation of the unaffected hemisphere with HF-rTMS may facilitate the emergence of this compensation and contribute to the recovery of swallowing function (Hamdy et al., 1998). However, our results in the subgroup analysis of overall swallowing function showed that the SMD of HF-rTMS (-0.70, *P* = 0.03) was smaller than LF-rTMS (-0.86, *P* = 0.0006); the subgroup analysis in PAS showed no significant effect of HF-rTMS (*P* = 0.09). At the same time, some of the studies (Park et al., 2013, 2017) included in this review used this model and did not observe any significant improvement in swallowing disorders. Therefore, it's rational to suspect that the mechanism of recovery from dysphagia after stroke is more complex than the interhemispheric inhibition model or the compensatory model.

In addition to this, a bimodal balance-recovery model has recently been proposed to describe the process of neuroplastic changes after stroke. This model incorporates the concept of “structural reserve”; if the brain has extensive damage and low structural reserve, then input from the unaffected hemisphere will be critical to replace the lost function; and conversely, if the structural reserve is high, then neural stimulation based on the interhemispheric inhibition model may be more appropriate (Sankarasubramanian et al., 2017). This provides a possible explanation for our conclusion. We found that bilateral stimulation was more effective than unilateral stimulation in some studies, and we considered that the use of transcranial magnetic stimulation in both hemispheres could produce a significant swallowing recovery effect by promoting plasticity in both hemispheres. We have found similar results in rTMS to improve other types of post-stroke dysfunction. For example, Jiang et al. (2020) observed higher effect values for the bilateral hemisphere compared to unilateral hemisphere stimulation in a meta-analysis of rTMS improvement of cognitive dysfunction. However, few studies have done the subgroup analyse according to degree of injury because the recruited patients had different degrees of brain injury. Raw data were also very difficult to obtain, so it was difficult for us to analyze them in subgroups according to different levels of injury. Future studies should be conducted in further subgroups according to different injury levels and time of stroke onset to explore the development of individualized treatment plans for patients.

In activity of daily living ability, we found significant improvement in the rTMS group, which is consistent with the studies of Liu et al. (2021) and Sui et al. (2021) rTMS can further improve hand function and cognitive function after stroke, thus further improving patients' motor ability and activity of daily living ability (Lefaucheur et al., 2020; Sharma et al., 2020).

In terms of treatment acceptability, the results of this study suggest that rTMS treatment was well-tolerated and there was no significant difference in the dropout rate between the rTMS treatment group and the control group. The reasons for follow-up failure were not mainly related to rTMS treatment, and no serious adverse effects were reported in any of the included trials. On the other hand, adverse reactions associated with rTMS were rare and mild, although patients in the rTMS-treated group were more likely to experience adverse reactions than the control group. The most common were transient headache and dizziness.

## Limitations

Several limitations should be considered when interpreting the results of the current study. First, the small sample size (12–29) of the included studies may limit the statistical power to detect the effects of rTMS on swallowing function in patients with post-stroke dysphagia. Second, there was considerable heterogeneity in the stimulation parameters (frequency, intensity and pulse) in the included studies. Therefore, the optimal stimulation parameters for rTMS are not clear. Third, our paper only performed subgroup analyses for frequency and stimulation site, but there was heterogeneity in the results of some of the subgroup analyses. The efficacy of rTMS may also be influenced by other parameters, such as brain injury severity and time to stroke onset.

## Conclusion

In conclusion, this meta-analysis study suggests that rTMS has a favorable effect on swallowing function in patients with post-stroke dysphagia. However, there are many parameters that can influence the efficacy, such as the frequency and the site of stimulation. Further research on the mechanism of rTMS and the setting of optimal parameters will be important for the development of this novel intervention in clinical practice.

## Data Availability Statement

The original contributions presented in the study are included in the article/supplementary material, further inquiries can be directed to the corresponding author/s.

## Author Contributions

Y-lX and SW were responsible for the literature screening and data extraction. SW, J-mJ, and Y-hX were responsible for risk of bias assessment. Y-lX and XC were responsible for statistical analysis and writing up the article. WQ and Y-xW were responsible for planning and guidance on this paper. All authors contributed to the article and approved the submitted version.

## Funding

This work was supported by Sichuan Medical Research Project Plan [Q18038]; Research and Development Project of Affiliated Hospital of North Sichuan Medical College [2021ZD014] and China Nanchong City-School Cooperative Scientific Research Special Fund [19SXHZ0103].

## Conflict of Interest

The authors declare that the research was conducted in the absence of any commercial or financial relationships that could be construed as a potential conflict of interest.

## Publisher's Note

All claims expressed in this article are solely those of the authors and do not necessarily represent those of their affiliated organizations, or those of the publisher, the editors and the reviewers. Any product that may be evaluated in this article, or claim that may be made by its manufacturer, is not guaranteed or endorsed by the publisher.
